# Patient satisfaction and tolerance of virtual reality rehabilitation in subacute ischemic stroke: a pilot study

**DOI:** 10.3389/fresc.2025.1660766

**Published:** 2025-08-26

**Authors:** Sarka Banikova, Alice Najsrova, Istvan Szegedi, Katerina Vitova, Iva Fiedorova, Jana Trda, Ondrej Volny

**Affiliations:** ^1^ Department of Rehabilitation and Sports Medicine, University Hospital Ostrava, Ostrava, Czechia; ^2^ Centre for Clinical Neurosciences, Faculty of Medicine, University of Ostrava, Ostrava, Czechia; ^3^ Department of Rehabilitation and Sports Medicine, Faculty of Medicine, University of Ostrava, Ostrava, Czechia; ^4^ Department of Neurology, University Hospital Ostrava, Ostrava, Czechia; ^5^ Department of Neurology, University of Debrecen, Debrecen, Hungary; ^6^ VR LIFE Ltd., Ostrava, Czechia

**Keywords:** virtual reality, stroke rehabilitation, patient satisfaction, neurorehabilitation, subacute stroke

## Abstract

**Background:**

Virtual reality (VR) rehabilitation represents a promising technological approach in post-stroke neurorehabilitation, offering immersive, engaging therapy environments. However, limited data exist on patient satisfaction and tolerance in clinical practice, particularly during the subacute phase of stroke recovery.

**Objective:**

To evaluate patient satisfaction and tolerance of VR rehabilitation in patients with subacute ischemic stroke and assess physiotherapist perceptions of treatment outcomes compared to conventional rehabilitation.

**Methods:**

A prospective pilot study was conducted from January 1, 2024, to December 31, 2024, at the Department of Neurology, University Hospital Ostrava, Czech Republic. Patients in the subacute phase of ischemic stroke (≤2 weeks post-stroke) underwent VR rehabilitation using the MDR-certified VR Vitalis® Pro system. Patient satisfaction was measured using the User Satisfaction Evaluation Questionnaire (USEQ) with individual question analysis. Physiotherapists assessed treatment outcomes on a 5-point scale compared to conventional rehabilitation. Vital signs were monitored pre- and post-intervention.

**Results:**

Nineteen patients (mean age 67.7 ± 11.2 years, 52.6% female) completed VR rehabilitation. The mean USEQ satisfaction score was 25.0 ± 6.8 points (range 7–30). High satisfaction (≥25 points) was achieved in 68.4% of patients, with only 5.3% reporting low satisfaction (<15 points). Individual question analysis revealed highest ratings for information clarity (4.63 ± 0.96) and perceived rehabilitation benefit (4.37 ± 1.12), with excellent tolerability as 63.2% reported no discomfort. Physiotherapists rated 31.6% of patients as showing better outcomes than expected with conventional therapy, while 52.6% showed similar outcomes and 15.8% showed worse outcomes. The mean number of VR sessions per patient was 4.2 ± 4.1. No serious adverse events were recorded.

**Conclusions:**

VR rehabilitation demonstrated high patient satisfaction and excellent tolerance in subacute stroke patients. Individual USEQ analysis revealed particularly strong acceptance for system clarity and rehabilitation benefit. These findings support the feasibility and acceptability of VR rehabilitation in clinical stroke care.

## Introduction

Stroke remains one of the leading causes of acquired disability worldwide, with approximately 33%–42% of patients requiring assistance with activities of daily living 3–6 months post-stroke (1). The burden of post-stroke disability encompasses sensorimotor deficits, cognitive impairment, speech disorders, and reduced quality of life, creating substantial socioeconomic challenges (2). Early and intensive rehabilitation during the subacute phase (typically defined as 7 days to 6 months post-stroke) is crucial for optimizing functional outcomes and promoting neuroplasticity (3).

Virtual reality (VR) technology has emerged as an innovative approach to neurorehabilitation, offering several theoretical advantages over conventional therapy (4). VR enables the creation of controlled, immersive environments that can provide intensive, task-specific training with real-time multimodal feedback (5). The technology facilitates motor learning through mirror neuron activation and promotes neuroplastic changes by engaging multiple brain regions simultaneously (6). Additionally, VR-based interventions are often perceived as more engaging and motivating than traditional rehabilitation exercises, potentially improving patient adherence and treatment outcomes (7).

Recent meta-analyses have demonstrated the efficacy of VR rehabilitation for various stroke-related impairments, including upper limb function, balance, gait, and cognitive performance (8–10). However, the majority of studies have focused on clinical effectiveness rather than patient-centered outcomes such as satisfaction and tolerance. Understanding patient perspectives is essential for successful implementation of VR technology in clinical practice, as user acceptance directly influences treatment engagement and long-term outcomes (11).

The MDR-certified VR Vitalis® Pro system represents a state-of-the-art rehabilitation platform specifically designed for neurological patients. Unlike conventional VR gaming systems, this medical device includes specialized modules for both upper and lower limb rehabilitation, with exercises designed to accommodate varying levels of motor impairment (1, 12). The system provides objective performance metrics and can be adapted for individual patient needs and therapeutic goals.

Despite growing evidence supporting VR rehabilitation efficacy, limited data exist on patient satisfaction and tolerance in real-world clinical settings, particularly during the early subacute phase of stroke recovery when patients may be most vulnerable to technology-related stress or fatigue. Furthermore, few studies have examined healthcare provider perspectives on VR rehabilitation outcomes compared to conventional therapy.

The primary objective of this pilot study was to evaluate patient satisfaction and tolerance of VR rehabilitation in patients with subacute ischemic stroke. Secondary objectives included assessing physiotherapist perceptions of treatment outcomes compared to conventional rehabilitation and documenting safety parameters during VR interventions.

## Methods

### Study design and setting

This prospective pilot study was conducted from January 1, 2024, to December 31, 2024, at the Department of Neurology and Department of Rehabilitation and Sports Medicine. The study was conducted in partnership with RBP Health Insurance Company and received approval from the institutional ethics committee University Hospital Ostrava, No 766/2023.

### Participants

Inclusion criteria comprised patients aged 18 years or older with ischemic stroke diagnosis confirmed by neuroimaging, in the subacute phase (within first month after stroke), with medically stable condition suitable for rehabilitation, ability to provide informed consent, and sufficient cognitive function to understand and follow VR instructions. Exclusion criteria included hemorrhagic stroke, severe cognitive impairment preventing VR comprehension, history of epilepsy or seizure disorders, severe visual impairment not correctable with glasses, motion sickness or vestibular disorders, unstable cardiovascular condition, and patient refusal to participate in VR therapy.

### VR rehabilitation intervention

All patients received VR rehabilitation using the MDR-certified VR Vitalis® Pro system (VR LIFE Ltd., Ostrava, Czech Republic) in addition to standard conventional rehabilitation. The VR system utilized Oculus Quest 2 VR headsets with wireless controllers, enabling both upper and lower limb exercises.

The virtual reality rehabilitation program comprised multiple therapeutic modules targeting different functional domains. These included bilateral upper limb coordination exercises (such as “Hanging laundry”), reach-and-grasp activities with adjustable difficulty levels (such as “Carrying mugs to shelves”), balance and postural control training, and cognitive-motor dual tasks that challenged patients' ability to perform simultaneous mental and physical activities.

The treatment protocol varied according to the phase of stroke recovery and hospital setting. Patients in the subacute phase of stroke (more than 24 h post-stroke) who were hospitalized in the stroke unit received daily VR therapy sessions. The total number of sessions during this phase of stroke care was determined by the duration of hospitalization. Patients who were subsequently transferred to the rehabilitation unit received an additional standardized protocol of 10 VR sessions delivered over a 14-day period. Consequently, the total number of VR therapy sessions per patient ranged from 4 to 13, depending on their clinical trajectory and length of stay across different care settings. Each VR session lasted approximately 10–20 min, with session frequency and content individualized based on patient tolerance and therapeutic goals. All sessions were supervised by qualified physiotherapists trained in VR system operation.

## Outcome measures

### Primary outcome

Patient satisfaction was assessed using the User Satisfaction Evaluation Questionnaire (USEQ), a validated 6-item instrument specifically designed for evaluating user satisfaction with virtual rehabilitation systems (13). The USEQ uses a 5-point Likert scale (1 = not at all, 5 = very much) and addresses key aspects of user experience including enjoyment, perceived success, system control, information clarity, comfort, and perceived rehabilitation benefit. The questionnaire includes one negatively worded item (Q5: “Did you feel discomfort during your experience with the system?”) which is reverse-scored for total score calculation. Total USEQ scores range from 6 (poor satisfaction) to 30 (excellent satisfaction), with scores ≥25 indicating high satisfaction, 15–24 indicating medium satisfaction, and <15 indicating low satisfaction.

### Secondary outcomes

• Physiotherapist assessment of treatment outcomes compared to conventional rehabilitation (5-point Likert scale: 1 = significantly worse than conventional therapy, 2 = slightly worse, 3 = similar to conventional therapy, 4 = slightly better, 5 = significantly better than conventional therapy)
• Vital signs monitoring (blood pressure and heart rate) before and after each VR session
• Number of VR sessions completed per patient
• Safety parameters and adverse events
• Treatment completion rates

### Data collection

Demographic information, stroke characteristics, and baseline clinical data were collected upon study enrollment. A specialized stroke nurse monitored vital signs immediately before and after each virtual reality rehabilitation session. Comprehensive physiotherapist assessments were conducted following completion of each patient's entire rehabilitation program. The User Satisfaction Evaluation Questionnaire (USEQ) was administered by an independent physiotherapist who was not affiliated with the research team, following completion of the full VR rehabilitation program.

### Statistical analysis

Descriptive statistics were used to summarize patient characteristics and outcome measures. Continuous variables are presented as mean ± standard deviation or median (interquartile range) as appropriate. Categorical variables are presented as frequencies and percentages. USEQ questionnaire responses were analyzed using descriptive statistics. Individual question responses were summarized using means, standard deviations, and frequency distributions for each response category (1–5 scale). The negatively worded question (Q5) was reverse-scored (6—original response) for inclusion in the total satisfaction score. Total USEQ scores were calculated as the sum of all six items (with Q5 reverse-scored) and categorized into satisfaction levels according to established criteria.

## Results

### Study population

During the study period, 30 patients (RBP health insurance members) were screened for eligibility, of whom 11 were excluded based on defined inclusion and exclusion criteria. Nineteen patients completed the VR rehabilitation program and were included in the final analysis.

### Baseline characteristics

The study population comprised 19 patients with a mean age of 67.7 ± 11.2 years (range: 46–86 years). The cohort was balanced by gender with 10 (52.6%) female and 9 (47.4%) male participants. All patients had ischemic stroke confirmed by neuroimaging. VR rehabilitation was initiated at a mean of 6 days post-stroke. The mean duration of conventional rehabilitation was 60 min per session. Patients completed a mean of 4.2 ± 4.1 VR sessions (range: 1–13 sessions) during their rehabilitation course (Table 1).

**Table 1:** Baseline patient characteristics and treatment parameters.

Characteristic	Value
Demographics	
Age, mean ± SD (range), years	67.7 ± 11.2 (46–86)
Female, *n* (%)	10 (52.6)
Male, *n* (%)	9 (47.4)
Stroke Characteristics	
Ischemic stroke	19 (100)
Treatment Parameters	
VR sessions per patient, mean ± SD (range)	4.2 ± 4.1 (1–13)
Conventional rehabilitation duration, minutes	60
Completed full rehabilitation course, *n* (%)	19 (100)

SD, standard deviation; VR, virtual reality.

### Primary outcome: patient satisfaction

The mean USEQ satisfaction score was 25.0 ± 6.8 points on the 30-point scale (range: 7–30 points). The distribution of satisfaction levels was (Table 2):

• High satisfaction (≥25 points): 13 patients (68.4%)
• Medium satisfaction (15–24 points): 5 patients (26.3%)
• Low satisfaction (<15 points): 1 patient (5.3%)

**Table 2:** Patient satisfaction and clinical outcomes.

Outcome measure	Value
Patient Satisfaction (USEQ)	
Mean satisfaction score ± SD (range)	25.0 ± 6.8 (7–30)
High satisfaction (≥25 points), *n* (%)	13 (68.4)
Medium satisfaction (15–24 points), *n* (%)	5 (26.3)
Low satisfaction (<15 points), *n* (%)	1 (5.3)
Physiotherapist Assessment	
Better than conventional therapy (4–5), *n* (%)	6 (31.6)
Similar to conventional therapy (3), *n* (%)	10 (52.6)
Worse than conventional therapy (1–2), *n* (%)	3 (15.8)
Mean physiotherapist rating ± SD	3.2 ± 0.8
Safety Parameters	
Serious adverse events, *n*	0
Sessions discontinued due to intolerance, *n*	0
Patients completing full course, *n* (%)	19 (100)

SD, standard deviation; USEQ, user satisfaction evaluation Questionnaire; VR, virtual reality.

### Individual USEQ question analysis

Analysis of individual USEQ questions revealed specific aspects of user satisfaction with the VR system (Table 3). The highest-rated aspect was information clarity (Q4: “Is the information provided by the system clear?”) with a mean score of 4.63 ± 0.96, where 78.9% of patients rated this as “very much” clear (score 5). This was followed by perceived rehabilitation benefit (Q6: “Do you think that this system will be helpful for your rehabilitation?”) with a mean score of 4.37 ± 1.12, and enjoyment (Q1: “Did you enjoy your experience with the system?”) with 4.21 ± 1.40.

**Table 3:** Individual USEQ question responses and frequencies.

Question	Question text	Mean ± SD	Score 1 *n* (%)	Score 2 *n* (%)	Score 3 *n* (%)	Score 4 *n* (%)	Score 5 *n* (%)
Q1	Did you enjoy your experience with the system?	4.21 ± 1.40	2 (10.5%)	1 (5.3%)	1 (5.3%)	2 (10.5%)	13 (68.4%)
Q2	Were you successful using the system?	3.84 ± 1.30	2 (10.5%)	0 (0.0%)	5 (26.3%)	4 (21.1%)	8 (42.1%)
Q3	Were you able to control the system?	4.00 ± 1.49	3 (15.8%)	0 (0.0%)	2 (10.5%)	3 (15.8%)	11 (57.9%)
Q4	Is the information provided by the system clear?	4.63 ± 0.96	1 (5.3%)	0 (0.0%)	0 (0.0%)	3 (15.8%)	15 (78.9%)
Q5^a^	Did you feel discomfort during your experience with the system?	1.79 ± 1.27	12 (63.2%)	3 (15.8%)	1 (5.3%)	2 (10.5%)	1 (5.3%)
Q6	Do you think that this system will be helpful for your rehabilitation?	4.37 ± 1.12	1 (5.3%)	0 (0.0%)	3 (15.8%)	2 (10.5%)	13 (68.4%)

^a^ Q5 is a negatively worded question.

For total USEQ score calculation, responses are reverse-scored (6—original score).

The lowest-rated positive aspect was perceived success (Q2: “Were you successful using the system?”) with a mean score of 3.84 ± 1.30, where 26.3% of patients provided neutral responses (score 3), suggesting some patients experienced challenges in achieving their intended goals within the VR environment. Regarding tolerability, the discomfort question (Q5: “Did you feel discomfort during your experience with the system?”) demonstrated excellent results with a mean score of 1.79 ± 1.27 on the original scale. Notably, 63.2% of patients reported no discomfort at all (score 1), and only 5.3% reported significant discomfort (score 5), indicating high tolerability of the VR intervention in this patient population.

## Secondary outcomes

### Physiotherapist assessment

Physiotherapists rated patient outcomes compared to expected results with conventional rehabilitation as follows:

• **Better than conventional therapy (scores 4–5):** 6 patients (31.6%)
• **Similar to conventional therapy (score 3):** 10 patients (52.6%)
• **Worse than conventional therapy (scores 1–2):** 3 patients (15.8%)

Overall, 84.2% of patients achieved outcomes that were similar to or better than those expected with conventional rehabilitation alone. VR rehabilitation was well-tolerated across the study population. No serious adverse events related to VR intervention were recorded. All vital sign changes remained within clinically acceptable ranges, and no patients required session termination due to cardiovascular concerns.

## Discussion

This pilot study provides valuable insights into patient satisfaction and tolerance of VR rehabilitation in early subacute ischemic stroke patients. The findings demonstrate high levels of patient satisfaction and good tolerance of VR technology, supporting its feasibility for integration into clinical stroke rehabilitation programs.

The mean USEQ satisfaction score of 25.0 points represents strong patient acceptance of VR rehabilitation, with nearly 70% of patients achieving high satisfaction levels. This finding is particularly significant given that our study population consisted of older adults (mean age 67.7 years) who may be less familiar with digital technologies. The high satisfaction rates suggest that age alone should not be considered a barrier to VR rehabilitation implementation. The individual question analysis provides valuable insights into specific aspects of user satisfaction with VR rehabilitation. Question 4 (information clarity) received the highest mean rating (4.63 ± 0.96), with 78.9% of patients rating it as “very much” clear, suggesting the VR system provided comprehensible feedback and instructions. This finding is particularly important for older adults, as clarity of information is crucial for technology acceptance in populations that may be less familiar with digital interfaces. Conversely, Question 2 (perceived success) showed the lowest mean score among positive items (3.84 ± 1.30), with 26.3% of patients rating their success as neutral (score 3). This suggests that while patients generally enjoyed the experience and found it clear, some struggled with achieving their intended goals within the VR environment. This finding aligns with the variability in motor impairment levels among stroke patients and highlights the importance of individualized difficulty adjustment in VR rehabilitation systems. The discomfort question (Q5) demonstrated excellent tolerability, with 63.2% of patients reporting no discomfort at all (score 1) and only 5.3% reporting significant discomfort (score 5). This low discomfort rate is particularly noteworthy given concerns about motion sickness and visual fatigue in VR applications, especially among older adults who comprised our study population (mean age 67.7 years). The high scores for perceived rehabilitation benefit (Q6: 4.37 ± 1.12) with 68.4% of patients strongly agreeing the system would be helpful, support the clinical relevance of VR rehabilitation from the patient perspective. This patient-perceived benefit is crucial for treatment adherence and long-term engagement with rehabilitation programs. Only one patient (5.3%) reported low satisfaction, indicating that VR rehabilitation is acceptable to the vast majority of stroke patients in the subacute phase. This finding aligns with previous research suggesting that VR-based interventions are perceived as more engaging and motivating than traditional rehabilitation exercises (14). The immersive, game-like nature of VR therapy may help maintain patient interest and motivation throughout the rehabilitation process, potentially leading to improved adherence and outcomes (15).

The physiotherapist assessments provide important clinical validation of VR rehabilitation effectiveness. Nearly one-third of patients (31.6%) were rated as achieving better outcomes than expected with conventional therapy alone, while over half (52.6%) achieved similar outcomes. Only 15.8% of patients were rated as having worse outcomes, suggesting that VR rehabilitation rarely interferes with expected recovery patterns. These findings are consistent with recent meta-analyses demonstrating that VR rehabilitation produces outcomes that are comparable or superior to conventional rehabilitation for various stroke-related impairments (16, 17). The fact that 84.2% of patients achieved outcomes similar to or better than conventional therapy supports the clinical value of VR as an adjunctive rehabilitation tool.

The absence of serious adverse events and the stable vital sign profiles throughout VR sessions demonstrate the safety of VR rehabilitation in this patient population. Previous concerns about motion sickness, eye strain, or cardiovascular stress appear to be minimal in properly supervised clinical settings with medical-grade VR equipment (18, 19). The MDR certification of the VR Vitalis® Pro system likely contributes to its safety profile compared to consumer-grade VR devices.

These results have several important implications for clinical stroke rehabilitation. VR rehabilitation demonstrated feasibility and good acceptance in early subacute stroke patients, even in older populations with a mean age of 67.7 years. When properly implemented with medical-grade equipment and trained supervision, VR rehabilitation appears safe for subacute stroke patients, as evidenced by the absence of serious adverse events in our pilot cohort. The positive physiotherapist assessments suggest that VR can be effectively integrated into existing rehabilitation programs without compromising clinical outcomes, with 84.2% of patients achieving results similar to or better than conventional therapy alone. Furthermore, the high satisfaction scores indicate that VR rehabilitation aligns well with patient preferences and may enhance the overall rehabilitation experience through its engaging and motivating nature.

Our findings align with recent research evaluating user satisfaction in VR-based neurorehabilitation. Roussou et al. (20) investigated the suitability, usability, and safety of fully immersive VR applications in stroke patients using the broader Suitability Evaluation Questionnaire (SEQ), reporting overall positive acceptance with scores of 61 (55–63) out of 65 points in only four patients during early rehabilitation phases. While their study utilized the more comprehensive SEQ questionnaire covering multiple usability dimensions, our focused approach using the USEQ allowed for specific assessment of satisfaction components most relevant to clinical implementation. The USEQ's targeted design for virtual rehabilitation systems provides more actionable insights for healthcare providers, as individual item analysis reveals specific aspects influencing patient acceptance, such as perceived therapeutic benefit (Q6) and system control (Q3), which directly inform clinical practice modifications. Similar to Roussou et al.'s findings of minimal adverse effects and good tolerance, our study demonstrated excellent safety profiles with high satisfaction scores, reinforcing the feasibility of VR integration in subacute stroke rehabilitation. The consistency between studies using different assessment tools strengthens the evidence base for VR rehabilitation acceptance across diverse patient populations and technological platforms, supporting broader clinical implementation while highlighting the importance of questionnaire selection based on specific research objectives and clinical information needs.

### Study limitations

Several limitations should be acknowledged when interpreting these results. This pilot study included a relatively small sample (*n* = 19), limiting the generalizability of findings and precluding robust statistical comparisons. The single-center design, conducted at a specialized academic medical center, may limit external validity to other clinical settings with different resources and patient populations. The absence of a randomized control group receiving only conventional rehabilitation limits our ability to make definitive comparisons of clinical effectiveness. Patients who agreed to participate in VR rehabilitation may have been more technology-accepting than the general stroke population, introducing potential selection bias. The study assessed immediate satisfaction and tolerance but did not evaluate long-term outcomes or sustained engagement with VR technology beyond the acute rehabilitation period. Additionally, the number and duration of VR sessions varied among patients based on individual clinical needs, making it difficult to establish optimal dosing parameters for future standardized protocols.

### Recommendations for clinical implementation

Based on our findings, several recommendations emerge for healthcare providers considering VR rehabilitation implementation. Adequate training of rehabilitation staff in VR technology operation and safety protocols is essential for successful program implementation. While age alone should not exclude patients, careful assessment of cognitive function and technology acceptance is important for optimal patient selection. VR rehabilitation programs should be tailored to individual patient needs, preferences, and tolerance levels to maximize therapeutic benefit and satisfaction. Continuous monitoring of vital signs and patient comfort during VR sessions is recommended, particularly during initial sessions when patients are adapting to the technology. Finally, VR rehabilitation appears most effective when used as an adjunct to, rather than replacement for, conventional rehabilitation techniques, suggesting that hybrid approaches may optimize patient outcomes.

## Conclusions

This pilot study demonstrates that VR rehabilitation using MDR-certified equipment is well-tolerated and highly satisfactory for patients in the early subacute phase of ischemic stroke. With 68.4% of patients achieving high satisfaction scores and 84.2% showing clinical outcomes similar to or better than conventional rehabilitation, VR technology appears to be a valuable adjunct to standard stroke rehabilitation protocols. Individual question analysis revealed particularly high acceptance for system clarity and perceived rehabilitation benefit, while excellent tolerability was demonstrated with minimal reported discomfort. The absence of serious adverse events and high treatment completion rates support the safety and feasibility of VR implementation in clinical practice. These findings provide important preliminary evidence for larger trials and support the integration of VR rehabilitation into comprehensive stroke care programs.

## Data Availability

The original contributions presented in the study are included in the article/Supplementary Material, further inquiries can be directed to the corresponding author.
